# Enhancing learning from evaluations in newborn and child health

**DOI:** 10.1136/bmjgh-2025-019663

**Published:** 2025-06-12

**Authors:** Claire Blacklock, Andrew Clarke, Sebastian Taylor, Marion Lynch, Arooj Sahir, Stavros Petrou, Junior Mudji, Shobhana Nagraj, Katherine Kalaris, Soren Kudsk-Iversen, Adam Harnischfeger, Claire Allen, Sarah Williams, Prisca Benelli, Claire M Keene, Desire Habonimana, Neil Jefferies, Tanya Marchant, Louise Tina Day, Mike English

**Affiliations:** 1 Health Systems Collaborative, University of Oxford Nuffield Department of Medicine, Oxford, England, UK; 2 Save the Children UK, London, UK; 3 Division of Health Research, Lancaster University, Lancaster, England, UK; 4 RCPCH Global, Royal College of Paediatrics and Child Health, London, UK; 5 Global Health Partnerships, London, UK; 6 Kings Global Health Partnerships, King’s College London, London, England, UK; 7 Nuffield Department of Primary Care Health Sciences, University of Oxford Nuffield Department of Medicine, Oxford, UK; 8 Department of Family Medicine and Primary Care, Protestant University of Congo, Kinshasa, Kinshasa, Congo (the Democratic Republic of the); 9 Department of Public Health and Primary Care, University of Cambridge, Cambridge, England, UK; 10 Oxford University Hospitals NHS Foundation Trust, Oxford, England, UK; 11 Oxford Policy Management, Oxford, England, UK; 12 Evidence Aid, Oxford, England, UK; 13 Faculty of Medicine, Centre de Recherche Universitaire en Santé (CURSA), University of Burundi, Bujumbura, Bujumbura Mairie, Burundi; 14 Oxford Bodleian Libraries, University of Oxford, Oxford, England, UK; 15 Infectious Disease Epidemiology & International Health, London School of Hygiene & Tropical Medicine, London, England, UK

**Keywords:** Health systems evaluation, Child health

**SUMMARY BOX**

- Operational learning from projects and programmes in newborn and child health in the international non-governmental organisations (INGOs) and the charity sector is vast. However, it is currently often not fully shared or used.
- A participatory action research event brought together 29 UK-based individuals from operational and academic sectors to identify and discuss concerns and recommendations for enhancing learning from project and programme evaluations and inform future dialogue with country-level partners.
- To enhance learning from evaluations of projects and programmes, the broader influences on how knowledge generation is approached must be addressed, including the role of institutional and governmental funding partners, and a ‘fear of failure’ culture in the INGO and charity sectors. Localisation of evaluation activities must be supported within the wider informational landscape, including through effective partnerships. Lastly, transformation of systems to enable better access to findings and reports of evaluations is needed.

## Introduction

 International non-governmental organisations (INGOs) that partner with national healthcare systems are well-positioned to contribute rich learning around implementation, and often influence health policy, guidelines and practice development.^1 2^ Learning presents an area of cross-over between programmes and academia, with findings useful to both parties, who may also directly collaborate on some projects. However, though guidance for designing and conducting evaluations is well-developed and useful learning can be achieved,^3^,^5^ multiple challenges are often faced. More effective learning could reduce waste and unnecessary duplication within and between projects, bringing additional benefits and fewer unintended harms to communities.^6^

We convened a participatory working group consisting of individuals working in UK-linked INGOs and academia.^7^ The aim was to bring together individuals predominantly focused on newborn and child health programmes, but with different experiences and backgrounds, to discuss and identify priority challenges and opportunities associated with learning, particularly from evaluations within charitable programmes (box 1). Recognising that this initial work included only UK-linked INGOs and academics, and that such conversations must be more inclusive, we offer initial thematic areas of discussion and recommendation in this commentary to inform such future dialogue.

Box 1 **Participatory action research in-person event: process**

Participants from INGOs and charities, and those with experience/skills relevant to conducting, using or appraising evaluations in healthcare, particularly newborn and child health (eg, academics, healthcare workers, consultants, etc) were invited. Consenting participants attended the in-person event at their own expense and were identified by snowballing through personal networks and the grey literature, including organisational websites.

Participants were invited to complete a prereading exercise, then attend a full-day, in-person event. A total of 29 participants attended the in-person meeting, including those working for INGOs, charities, academia, consultancies, libraries and knowledge mobilisation groups.

The participatory format included short presentations, facilitated ‘fishbowl’ discussions, small group and whole-group discussions and informal networking over lunch and extended refreshment breaks. Visual cues and prompts for discussion (eg, examples of evaluation guidelines, responses arising from prereading) were displayed in the space throughout the day. The participatory action research was approved by OXTREC (Reference: 556–23).

In addition to the responses to the prereading materials, data collected during the in-person event included: slides from short presentations, notes taken during the event by four parallel notetakers, group discussion outputs (eg, flip chart diagrams and notes) and back-up audiorecordings of small group discussions to support notetaking. Data were analysed thematically. All participants were invited to contribute to this manuscript.

## Outputs

There were three main areas of discussion: (1) challenging broader influences shaping knowledge generation; (2) localising monitoring and evaluation activities; and (3) transforming access to evaluation findings and reports.

### Challenging broader influences shaping knowledge generation

Participants expressed that, while individuals and organisations are often keen to openly discuss failures as well as successes, this is not the norm because the political economy of development assistance funding and negative portrayals in the media lead to a ‘fear of failure’. There is a perceived tension between the public good of learning from stories of failure on the one hand, and the risk to individual organisations if such sharing results in reputational damage in the eyes of the general public, and then loss of funds. This can result in evaluations only being shared internally within an organisation, for example, leading to lost learning opportunities for the broader sector and academia. There is also a risk of manipulation of indicators to reduce the perceived degree of failure, compromising learning.

Different actors were felt to value different types of information. Moreover, the project context was asserted as being complex. Specific details of projects and implementation contexts, including human behaviours, were considered vital for understanding what works for whom, and in what circumstances. However, these details were felt to be often missed from evaluations, undermining measurement and understanding of what makes a ‘good project’.

Discourse around the deemed ‘failure’ of projects and programmes was felt to require reorientation to permit a more constructive and rounded approach to learning. For example, a project deemed to have ‘failed’ in its objectives (in some cases from unrealistic expectations) may still contribute rich knowledge to inform future interventions. However, INGOs and charities, as well as bilateral funding partners and governments, are subject to external pressures, domestic politics and shifts in public attitudes. Therefore, reorientation is likely to require considerable commitment, as well as clear support for honest learning to be a routine expectation of development assistance. Organisations will need courage to share evaluations for the purposes of learning. Indeed, leaders in the sector must champion learning as part of organisational strategies. Critically, there is a key role here for those funding INGOs to facilitate a psychologically safe^8^ environment, through how learning is positioned, framed and valued. Further support for sector-wide collaboration around a strengthened learning culture could be implemented through initiatives such as position statements, charters, codes of practice and could include tools such as maturity matrices, that reflect the ability of systems to generate and absorb learning.^9^

### Localising monitoring and evaluation activities

Participants felt INGO monitoring and evaluation activities typically remained parallel to local information and data systems. While not all data for evaluation and learning are appropriate to be collected by routine health (management) information systems (RHIS/HMIS), investment in RHIS is important. Likewise, essential skills development at community level (eg, critical thinking, reflection) could be supported by project-level initiatives such as team sharing platforms and local networks of practice. Indeed, empowerment of project staff and local partner organisations might counter preconceptions that data collection (and its use) is the remit solely of researchers, or INGO Head Office staff, or valued primarily for funding partner reporting purposes. Such a commitment should be prioritised to improve information quality and support effective partnerships.^10^

Responsiveness and adaptability of projects can be enhanced through local leadership and more effective continuous learning at project level. Higher-level project and programme evaluations would also become more efficient and valuable when anchored in more meaningful and accurate data, stepping beyond funding partner reporting requirements to better reflect the priorities, values and power dynamics of government and community. Indeed, constructing a ‘true’ theory of change is likely to yield enormous value—beyond a more rushed and superficial version, without sufficient depth, scope, community involvement, put together to primarily fulfil funding requirements.

Hearing and valuing community voices require a safe learning culture, to mitigate individual risks from granular data, avoid blame, permit challenge and encourage accuracy and quality. Monitoring and evaluation activities should maximise project staff ownership and use of data, without overload of individuals (eg, healthcare workers). Successful partnerships must be grown, with delegation of ‘the right task to the right group’. Academic involvement in projects must be considered carefully, as despite expertise and ability to publish, researchers may ask the wrong questions, take a long time, and hold different priorities to operational programme staff.

### Transforming access to evaluation findings and reports

Participants expressed that INGOs and charities faced many practical day-to-day challenges around sharing learning from evaluations, both internally and externally. Internal processes for sharing findings of evaluations within teams and organisations can be fragile and reliant on continuity of personnel. Gaps between projects, or staff turnover, contribute to the loss of learning from one project to the next. Bureaucracy and time constraints mean that evaluation reports can get ‘filed’ online, without adequate sharing. Furthermore, processes may restrict information circulation and sharing, whether for legitimate reasons such as protecting vulnerable groups, or less justifiably due to more complex conflicts or fears of negative impacts on fundraising or relationships. Internally, teams may also fear exposure from sharing reports with other teams. In addition, staff busy with other projects may not have ‘headspace’ to read long reports with inaccessible jargon, which are sometimes perceived to have limited value for practical application. This can lead to systematic exclusion from learning of those on the frontline of projects and within communities. Lateral links between different projects, as well as links between old and new projects, would help foster a sense of wider team and learning community, supported by innovations such as internal sharing events, already initiated by some INGOs and charities.

External sharing of evaluation findings beyond INGOs and charities can be challenging. The rigid requirements (and costs) and lens of academic journals deter and preclude the publication of many programme and practice-related learning papers. Without academic affiliation, INGOs lack access to many journals. Organisational websites and other alternative online publishing formats may require time-consuming navigation to locate downloadable reports, if available at all. Indeed, many evaluation reports from INGOs and charities are never made publicly accessible.

There are some platforms in which INGOs can publish evaluations. For instance, the ALNAP Evaluation Mapper is the sector’s largest library of resources on Humanitarian Learning.^11^ Large organisations, like Save the Children and Médecins Sans Frontières (MSF),^12 13^ also have dedicated websites where evaluations are accessible to external stakeholders—although sometimes located separately to the main organisation website—with additional reports being uploaded on sections only accessible to staff. As evaluation information is often dispersed, an appropriate mechanism by which to publish, share, store and find reports of evaluations of projects and programmes in the INGO and charity sectors is needed. We propose such a platform should: (1) be open access, (2) be searchable, (3) include a register of projects, (4) include mechanisms for peer commentary where appropriate and (5) include mechanisms for ethical inputs where appropriate. Indeed, facilitating a process of open peer commentary may further encourage sharing of critical thinking from diverse perspectives.

The addition of lay summaries is also likely to play an important role (possibly harnessing AI technology, while safeguarding for associated biases).^14^ Moreover, intermediary knowledge mobilisation organisations could extend their existing role, to broker learning in this space.^15^ A network or consortium could also help to share findings between organisations and groups.

## Conclusions

Enhancing learning from evaluations of projects and programmes in newborn and child health requires transformation within the operational INGO and charity sectors, akin to that following the open access movement in academia.^10^ The broader influences shaping knowledge generation, including institutional and governmental funding partners and ‘fear of failure’ culture, need to be reorientated. Localisation of monitoring and evaluation activities should be supported with effective partnerships. Lastly, access to the findings and reports of evaluations should enable useful learning to be accessible and searchable for all.

## Data Availability

Data are available upon request.
